# Annotations

**Published:** 1908-12-19

**Authors:** 


### Annotations.

### Methods of Hysteria.

It would seem that the charge which is brought against modern civilisation of producing hypersensitiveness to stimulus is not without some basis of truth. Day by day we have brought before our notice cases of ungovernable hysteria, which a sentimental press characterises as enthusiasm for a good cause. Because there have been hysterical fanatics who have succeeded in obtaining their requests, there is not therefore a corollary that hysteria spells success; far from it. As heterogeneity is increasing, and life becoming more complex, we are beginning to recognise that the ills of the mind, however subtle they may be, are just as serious as the more obvious corporeal lesions, and are even more in need of judicious treatment. We cannot doubt that the present exhibitions of over-excitability, particularly among the women and weaker men of our community, are an indication of some serious deficiency in mental balance. We are for ever being told that it is brain that rules the world, and that muscle is almost a disgrace. This exaggerated view of brain is obviously producing effects which are of no very promising character. There is this absurd tendency to deify the neurotic, and to see in every hysterical and unrestrained woman the champion of a cause—in fact, a kind of modern Boadicea. But there can be no doubt that this hysteria is bad for the country at large, as well as for the individuals, who know not what they do. It is not a good sign in any country to have irresponsible women using dogwhips in a moment which, were they of lower social status, would secure for them the punishment meted out to brawlers or the restrictions placed upon the weak-minded. Nor can we view with satisfaction the adherence of certain individuals of the opposite sex, whose function seems to be that of "complemental males" so often noticed among Crustacea. We desire, above all things, methods of manliness and sobriety, and we are inclined to think that the time has come for some serious notice to be taken of a symptom which to us is fraught with grave significance. To each climate its characteristics. We can extenuate such hysterical outbursts among southern races, where emotionalism must always play so large a part, but we deprecate the transformation of the steadier northern solidnesses into promiscuous and unreasoning hysteria. The present scenes tend to prove almost conclusively that biological and physiological differences of sex are becoming more and more emphasised. Civilisation produces divergence, not appropinquity. The influence of woman as an individual, as a power behind man, will never be diminished so long as it is recognised that her sphere is not splendid isolation. There is nothing normal in the present manifestations, nor do we think that abnormal woman has any place in the Constitution, save a pleasant house of restraint, where, under care and proper treatment, she may regain her lost self-control.

### The Bradshaw Lecture.

In our modern eagerness to adopt the latest "new thing" in medical or surgical treatment, prompted by the fear of being otherwise considered unprogressive, there is a definite risk of methods which are valuable but not brand-new falling into a certain amount of disuse. It is one of the thoroughly useful ends served by certain annual lectureships that they provide opportunities for retrospect into the past, and the recent Bradshaw lecture of Sir W. Watson Cheyne is a forceful case in point. The lecturer's chief argument is that the pendulum of surgical opinion on the treatment of wounds, in swinging away from reliance entirely on antiseptics, has overshot the golden mean, and has oscillated too far towards simple aseptic sterilisation. He does not regard with horror the possibility of dilute antiseptic solutions entering an operation wound, for he is convinced that the weak solutions now in common use at operations are so nearly harmless to the tissues that the good which they do outweighs the slight disadvantages. He objects particularly to three of the practices of the present aseptic school of surgeons. Thus dressings which are merely sterilised without containing antiseptics, the absence of antiseptic solutions in which the surgeon may rinse his hands during an operation, and the substitution of gauze wicks for drainage tubes fall under condemnation. On the second of these points it is perhaps permissible to point out that there is a great difference, according as gloves are or are not worn; in the latter case sterilised water is surely sufficient. On the first, and especially on the third, indictments it is probable that the large majority of practising surgeons, in which term we include the main mass of the profession throughout the kingdom, will be distinctly in agreement with Sir Watson Cheyne. It is not always remembered, as it should be, by surgical teachers in the medical schools of our large towns that what is safe and practicable in the perfectly equipped theatres of a fine hospital where expense is no object (to the surgeons), is not necessarily best adapted to the exigencies of rough-and-tumble accident surgery in general practice; nor even for more deliberate and eclectic operative work when the various assistants, male and female, may not have had thorough training in the application of the principles of applied bacteriology. It is right to hold up a lofty ideal to students, but it is also right to show them a practical compromise for every-day use. Professor Cheyne thus maintains his position as the chief exponent of the original principles of Listerism, and indeed of the actual antiseptic practices of Lord Lister himself, after the abolition of such early crudities as the carbolic spray.

